# Substrate-inducible and antibiotic-free high-level 4-hydroxyvaleric acid production in engineered *Escherichia coli*

**DOI:** 10.3389/fbioe.2022.960907

**Published:** 2022-08-09

**Authors:** Chandran Sathesh-Prabu, Rameshwar Tiwari, Sung Kuk Lee

**Affiliations:** School of Energy and Chemical Engineering, Ulsan National Institute of Science and Technology (UNIST), Ulsan, South Korea

**Keywords:** 4-hydroxyvaleric acid, antibiotic-free, promoter, inducible expression, levulinic acid

## Abstract

In this study, we developed a levulinic acid (LA)-inducible and antibiotic-free plasmid system mediated by HpdR/P_*hpdH*_ and *infA*-complementation to produce 4-hydroxyvaleric acid (4-HV) from LA in an engineered *Escherichia coli* strain. The system was efficiently induced by the addition of the LA substrate and resulted in tight dose-dependent control and fine-tuning of gene expression. By engineering the 5′ untranslated region (UTR) of *hpdR* mRNA, the gene expression of green fluorescent protein (GFP) increased by at least two-fold under the *hpdH* promoter. Furthermore, by evaluating the robustness and plasmid stability of the proposed system, the engineered strain, IRV^750f^, expressing the engineered 3-hydroxybutyrate dehydrogenase (3HBDH^∗^) and formate dehydrogenase (*Cb*FDH), produced 82 g/L of 4-HV from LA, with a productivity of 3.4 g/L/h and molar conversion of 92% in the fed-batch cultivation (5 L fermenter) without the addition of antibiotics or external inducers. Overall, the reported system was highly beneficial for the large-scale and cost-effective microbial production of value-added products and bulk chemicals from the renewable substrate, LA.

## Introduction

Owing to concerns regarding environmental pollution by the excessive use of fossil fuels and the ongoing depletion of fossil fuel resources, various biological processes have been developed to produce bulk chemicals using eco-friendly and renewable substrates (Kim D. et al., 2019; Hazeena et al., 2019; Sathesh-Prabu et al., 2019; Zhang et al., 2021). Advances in synthetic biology and metabolic engineering have resulted in the development of efficient microbial cells, such as *Escherichia coli*, to produce biochemicals with wide applications in the medical, environmental, and industrial sectors based on a renewable biomass feedstock (Lee et al., 2019). The success of a high-performance microbial host depends on its ability to express and control the pathway gene(s) required for improved biochemical production. As high-degree (∼100 s) gene dosage can be attained using appropriate plasmids in host cells, synthetic biology and metabolic engineering rely on plasmid-based biochemical production systems (Kang C. W. et al., 2018). Maintaining a plasmid inside the host cell requires selective pressure, which is typically achieved through the use of an antibiotic molecule (Saleski et al., 2021). However, this approach has limitations, such as antibiotic cost and the dissemination of antibiotic resistance, particularly in large-scale cultures, in addition to its low efficiency induced by its gradual degradation (Rosano and Ceccarelli, 2014). Antibiotic-free plasmid maintenance systems have been developed by deleting essential genes from the host and complementing them with plasmids (Hägg et al., 2004; Kang C. W. et al., 2018). One of the essential target genes is *infA*, which encodes the translation initiation factor, IF-1 (Kang C. W. et al., 2018).

Inducible promoter systems are widely used to control gene expression, which is a crucial requirement in synthetic biology for the efficient expression of the desired pathway (Keasling, 1999). Many different promoter systems that respond to various stimuli, including sugars and their analogs, metabolic intermediates, salts, metals, antibiotics, oxidative stress, and temperature, have been developed for recombinant protein expression in *E*. *coli* (Kelly et al., 2016; Marschall et al., 2017; Sathesh-Prabu et al., 2020). Promoter expression induction by the addition of chemical inducers such as isopropyl-β-d-1-thiogalactopyranoside (IPTG) is considered the most efficient method (Makrides, 1996; Briand et al., 2016). However, this approach has limitations, including the requirement for the monitoring of cell growth for the addition of chemical inducers at the optimal cell density. Furthermore, owing to its toxicity and cost, this approach is not feasible for industrial scale-up (Makrides, 1996; Briand et al., 2016).

4-Hydroxyvaleric acid (4-HV), a versatile compound possessing both carboxy and hydroxy moieties, can be converted into useful products, including biodegradable and biocompatible polyesters, fine chemicals, and pharmaceuticals (Gorenflo et al., 2001; Martin and Prather, 2009; Muzaiyanah and Amirul, 2013; Sathesh-Prabu and Lee, 2019). 4-HV can be used as a monomer for the production of polyhydroxyalkanoates (PHAs) (Gorenflo et al., 2001; Cha et al., 2020; Kim and Lee, 2022); it provides biopolymers (polyesters) with better physical and mechanical properties upon polymerization with other hydroxy acids (Gorenflo et al., 2001; Yu, 2010). The conventional chemical synthesis of 4-HV as an intermediate or via the depolymerization of intracellularly produced PHA has various shortcomings, including low yield, the use of harsh conditions, metal catalysts, and organic solvents, the need for chemical modification, and incomplete depolymerization (Martin and Prather, 2009). 4-HV can be produced from levulinic acid (LA) (Martin and Prather, 2009; Yeon et al., 2013; Sathesh-Prabu and Lee, 2019; Moon et al., 2021). We previously engineered the *lva* operon involved in LA catabolism in *Pseudomonas putida* KT2440 (Rand et al., 2017) and produced 50 g/L of 4-HV by overexpressing the heterologous acyl-CoA thioesterase gene, *tesB*, under the LA-inducible LvaR/P_*lvaA*_ expression system, with a molar conversion of 97% from LA in shake-flask cultures (Sathesh-Prabu and Lee, 2019). We also reported the biological synthesis of 4-HV in an *E. coli* strain expressing the engineered 3-hydroxybutyrate dehydrogenase (3HBDH*) (Yeon et al., 2013), which produced 100 g/L of 4-HV under optimized growth conditions from LA by co-expressing the NADH regeneration system catalyzed by NAD^+^-dependent formate dehydrogenase (*Cb*FDH) of *Candida boidinii* in fed-batch (5 L fermenter) cultivation using an IPTG-inducible expression system (Kim D. et al., 2019). Of note, formate was used as the co-substrate to regenerate NADH.

We recently reported that the 3-hydroxypropionic acid (3-HP)-inducible HpdR/P_*hpdH*_ system could be induced by LA and evaluated its efficiency in biotechnologically-relevant hosts, including *P*. *putida* KT2440 (Sathesh-Prabu et al., 2021b) and *Methylorubrum extorquens* AM1 strains (Sathesh-Prabu et al., 2021a). LA is a sustainable molecule that can be obtained from renewable cellulosic biomass via acid-catalyzed dehydration and hydrolysis (Hayes and Becer, 2020; Moon et al., 2021). Substrate-inducible expression systems have attracted attention for the microbial synthesis of biochemicals, as they do not require expensive chemical inducers.

**FIGURE 1:**
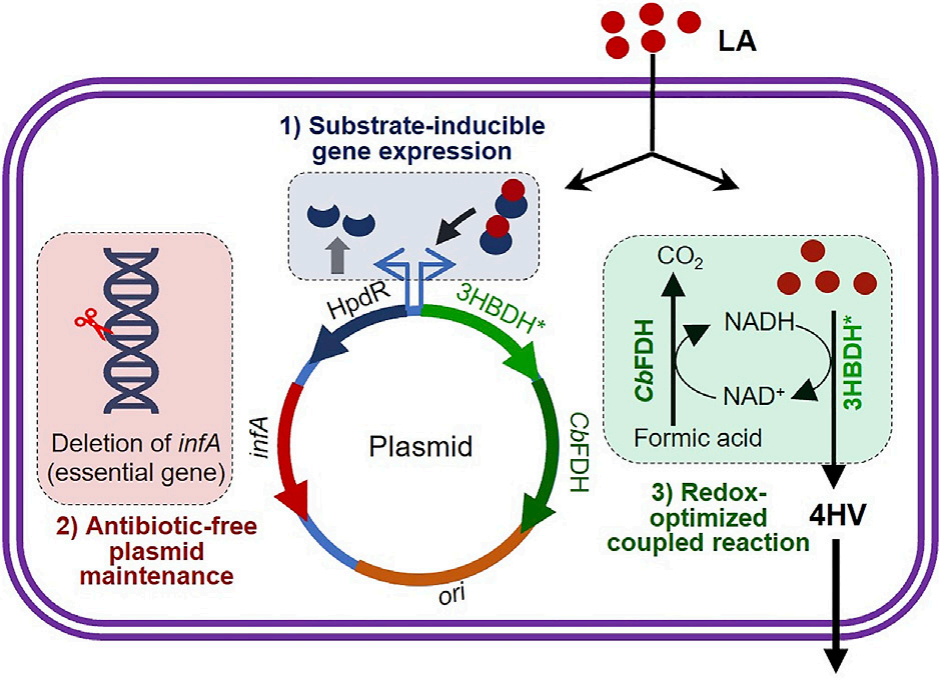
Schematic illustration of the three strategies applied to achieve substrate-inducible and antibiotic-free 4-hydroxyvaleric acid production in *Escherichia coli*. *infA* (essential gene) was deleted from the genomic DNA of *E. coli*. An LA-inducible HpdR/P_*hpdH*_ expression system was constructed, which included the functional expression of *infA*. 4-HV was produced under redox–process optimized conditions. Refer to the text for more details. LA, levulinic acid; 4-HV, 4-hydroxyvaleric acid; 3HBDH*, engineered 3-hydroxybutyrate dehydrogenase; *Cb*FDH, formate dehydrogenase.

We aimed to develop an *infA*-based antibiotic-free plasmid and LA-inducible HpdR/P_*hpdH*_ system for the sustainable production of the industrially-relevant chemical, 4-HV, in an engineered *E. coli* strain using 3HBDH* and *Cb*FDH from the low-cost renewable substrate, LA, under antibiotic- and external inducer-free conditions (Figure 1). Before the production studies, we evaluated the efficiency of the constructed expression system by analyzing green fluorescent protein (GFP) expression as a quantitative reporter of promoter activity. We also optimized the gene expression system by engineering the 5′-untranslated region (UTR) of *hpdR* mRNA for the high expression of pathway genes.

## Materials and methods

### Microbial strains and plasmids

The wild-type (WT) *E. coli* MG1655 strain was used as the parental strain for all genetic modifications and the analysis of the efficiency of the constructed expression system. The *E. coli* DH10B strain was used for cloning. The strains and plasmids used and constructed in this study are listed in Table 1.

**TABLE 1:** Strains and plasmids used in this study.

Strains and plasmids	Description	References
**Strains**		
*E. coli* DH10B	Cloning host (F^−^ *mcrA* Δ(*mrr*-*hsdRMS*-*mcrBC*) φ80*lacZ*ΔM15 Δ*lacX74 recA1 endA1 araD139* Δ(*ara*-*leu*)*7,697 galU galK* λ^–^ *rpsL*(Str^R^) *nupG*)	Lab stock
WT	*E. coli* K-12 MG1655 F^−^λ^–^ *ilv*G^–^ *rfb*-50*rph-1*	Blattner et al. (1997)
MG_*infA* ^−^	WT with ∆*infA*	This study
IRF	MG_*infA* ^−^ harboring pHRH_IA_eGFP^+^	This study
IRV	MG_*infA* ^−^ harboring pHRH_IA_3HBDH*/*Cb*FDH	This study
RV	WT harboring pHRH_3HBDH*/*Cb*FDH	This study
ILF	MG_*infA* ^−^ harboring pLIL_IA_eGFP^+^	This study
ILV	MG_*infA* ^−^ harboring pLIL_IA_3HBDH*/*Cb*FDH	This study
IRF^10f^	MG_*infA* ^−^ harboring pHRH^10f^_IA_eGFP^+^	This study
IRF^100f^	MG_*infA* ^−^ harboring pHRH^100f^_IA_eGFP^+^	This study
IRF^250f^	MG_*infA* ^−^ harboring pHRH^250f^_IA_eGFP^+^	This study
IRF^500f^	MG_*infA* ^−^ harboring pHRH^500f^_IA_eGFP^+^	This study
IRF^750f^	MG_*infA* ^−^ harboring pHRH^750f^_IA_eGFP^+^	This study
IRF^1Kf^	MG_*infA* ^−^ harboring pHRH^1Kf^_IA_eGFP^+^	This study
IRV^750f^	MG_*infA* ^−^ harboring pHRH^750f^_IA_3HBDH*/*Cb*FDH	This study
IRV^1Kf^	MG_*infA* ^−^ harboring pHRH^1Kf^_IA_3HBDH*/*Cb*FDH	This study
**Plasmids**		
pPROBE_P_*yqjFmut*__eGFP^+^	BBR1-*ori*, Km^R^, broad-host range expression vector	Kim et al. (2019b)
pHRH-eGFP^+^	pPROBE_HpdR/P_*hpdH*__eGFP^+^; LA-inducible	Sathesh-Prabu et al. (2021b)
pBbE6k_3HBDH*	pBbE6k_*rfp* with Δ*rfp*::3HBDH*, Km^R^	Kim et al. (2019a)
pBbB6a_*Cb*FDH	pBbB6a_*gfp* with Δ*gfp*::*Cb*FDH, Amp^R^	Kim et al. (2019a)
pBbE6k_RFP	ColE1-*ori*, carrying P_*LlacO1*_ and *rfp*, Km^R^	Lee et al. (2011)
pSIM5	pSC101^ts^- *ori*, Cm^R^, expressing λ-Red *gam*, *exo*, and *bet*	Datta et al. (2006)
pSIM5_*infA*	*infA* cloned into pSIM5	This study
pHRH_IA_eGFP^+^	*infA* cloned into pHRH-eGFP^+^; LA-inducible	This study
pHRH_IA_3HBDH*	3HBDH* cloned into pHRH_IA_eGFP^+^	
pHRH_IA_3HBDH*/*Cb*FDH	*Cb*FDH cloned into pHRH_IA_3HBDH*; LA-inducible	This study
pLIL_IA_3HBDH*/*Cb*FDH	HpdR/P_*hpdH*_ replaced with LacI/P_*LlacO1*_ in pHRH_IA_3HBDH*/*Cb*FDH; IPTG-inducible	This study
pLIL_IA_eGFP^+^	3HBDH*/*Cb*FDH replaced with eGFP^+^ in pLIL_IA_3HBDH*/*Cb*FDH; IPTG-inducible	This study
pHRH_3HBDH*/*Cb*FDH	eGFP^+^ replaced with 3HBDH*/*Cb*FDH in pHRH-eGFP^+^; LA-inducible	This study
pHRH^10f^_IA_eGFP^+^	pHRH_IA_eGFP^+^ with the UTR variant 10f	This study
pHRH^100f^_IA_eGFP^+^	pHRH_IA_eGFP^+^ with the UTR variant 100f	This study
pHRH^250f^_IA_eGFP^+^	pHRH_IA_eGFP^+^ with the UTR variant 250f	This study
pHRH^500f^_IA_eGFP^+^	pHRH_IA_eGFP^+^ with the UTR variant 500f	This study
pHRH^750f^_IA_eGFP^+^	pHRH_IA_eGFP^+^ with the UTR variant 750f	This study
pHRH^1Kf^_IA_eGFP^+^	pHRH_IA_eGFP^+^ with the UTR variant 1000f	This study
pHRH^750f^_IA_3HBDH*/*Cb*FDH	eGFP^+^ replaced with 3HBDH*/*Cb*FDH in pHRH^750f^_IA_eGFP^+^	This study
pHRH^1Kf^_IA_3HBDH*/*Cb*FDH	eGFP^+^ replaced with 3HBDH*/*Cb*FDH in pHRH^1Kf^_IA_eGFP^+^	This study

### Chemicals, enzymes, and culture conditions

All chemicals and media were purchased from Sigma-Aldrich (St. Louis, MO, United States). Restriction enzymes, DNA ligase, Q5 high-fidelity DNA polymerase, and the Gibson assembly cloning kit were purchased from New England Biolabs (Ipswich, MA, United States). LA was neutralized with 10 N NaOH and sterile filtered before use. The media, Luria–Bertani (LB; composition per liter: 5 g yeast extract, 10 g peptone, and 10 g NaCl) and terrific (TB; composition per liter: 12 g tryptone, 24 g yeast extract, 9.4 g potassium phosphate, dibasic; 2.2 g potassium phosphate, monobasic; and 4 g glycerol) broths were used for routine and high-cell density cultivation, respectively, at 37°C with shaking at 200 rpm. The media were supplemented with 30 μg/ml chloramphenicol (Cm) or 50 μg/ml kanamycin (Km), as required.

### Construction of the antibiotic-free and LA-inducible expression system

*infA* was deleted from the *E. coli* MG1655 strain using a λ-Red and FLP-protein-mediated site-specific recombination system, as described previously (Datsenko and Wanner, 2000), with a slight modification. First, a fragment containing *infA* and its native promoter was PCR-amplified from *E. coli* MG1655 genomic DNA. The fragment was then cloned into pSIM5 to generate pSIM5_*infA*. The pSIM5_*infA* plasmid contains the temperature-sensitive replication protein, RepA101, and the λ-Red recombinase genes under the P*L* promoter controlled by the cI857 temperature-sensitive repressor. Furthermore, the λ-Red system can be easily switched on at 42°C and off at 32°C (Datsenko and Wanner, 2000). The λ-Red system comprises three genes: *exo* (Exo), *bet* (Beta), and *gam* (Gam) for homologous DNA recombination (Murphy, 1998). This pSIM5_*infA* plasmid was used to complement *infA* and express the λ-Red system during chromosomal *infA* deletion. Finally, the strain, MG_*infA* ^−^ (MG_Δ*infA*), was generated. An LA-inducible HpdR/P_*hpdH*_ expression system was subsequently constructed, with the functional expression of *infA*. To construct pHRH_IA_eGFP^+^ (a plasmid containing eGFP^+^ under HpdR/P_*hpdH*_ and InfA), the *infA*-fragment was cloned into the *EcoR*I digested expression vector, pHRH_eGFP^+^ (Sathesh-Prabu et al., 2021b), a derivative of the pPROBE_P_*yqjFmut*__eGFP^+^ plasmid (pBBR1-*ori*, Km^R^: a broad-host-range expression vector) (Kim S. et al., 2019).

To produce 4-HV from LA under antibiotic-free and LA-inducible conditions, pHRH_IA_3HBDH*/*Cb*FDH was constructed by replacing *egfp* ^*+*^ in pHRH_IA_eGFP^+^ at the *Nde*I/*Hind*III sites with 3HBDH* to generate pHRH_IA_3HBDH*. Subsequently, *Cb*FDH was transcriptionally fused with 3HBDH* at the *Aat*II/*Hind*III sites to generate pHRH_IA_3HBDH*/*Cb*FDH. 3HBDH* and *Cb*FDH were PCR-amplified using pBbE6k_3HBDH* and pBbB6a_*Cb*FDH as templates. pHRH_3HBDH*/*Cb*FDH was also generated without *infA* by replacing *egfp* ^*+*^ with the 3HBDH*/*Cb*FDH genes in pHRH_eGFP^+^ at the *Nde*I/*Hind*III sites. To compare the efficiency of the constructed system, IPTG-inducible pLIL_IA_3HBDH*/*Cb*FDH was constructed by replacing HpdR/P_*hpdH*_ in pHRH_IA_3HBDH*/*Cb*FDH with LacI and the P_*LlacO1*_ fragment at the *Sac*I/*BamH*I sites. This fragment was amplified using the pBbE6k_RFP vector (Lee et al., 2011). Similarly, pLIL_IA_eGFP^+^ was constructed by replacing 3HBDH*/*Cb*FDH with eGFP^+^ at the *Nde*I/*Hind*III sites in pLIL_IA_3HBDH*/*Cb*FDH. The primers used in this study are listed in Supplementary Table S1. The constructed plasmids were transformed into electro-competent cells of the WT or MG_*infA* ^−^ strains. To prepare electro-competent cells, the strains were cultured overnight in LB and then subcultured (1:100) in 5 ml fresh LB. Following the cultivation of the strains for at least 2 h (optical density at 600 nm [OD_600_] = 0.3–0.5), the cells were chilled on ice for 20 min. The cells were then harvested via centrifugation at 16,000 × *g* for 1 min at 4°C, washed twice with cold sterile water, and resuspended in cold 10% glycerol. After the target plasmid was added to this suspension, electroporation (0.1 cm gap cuvette at 1.8 kV voltage) was carried out using a MicroPulser electroporator (Bio-Rad).

### Engineering of the 5′ UTR of HpdR

As HpdR is a transcriptional activator, its expression was modulated by engineering the 5′ UTR of *hpdR* mRNA to obtain different expression levels of pathway genes under the HpdR/P_*hpdH*_ system. *hpdR* mRNA UTRs exhibiting 10-, 100-, 250-, 500-, 750-, and 1000-fold higher translation levels relative to those in the native system were designed using the online UTR designer program (https://sbi.postech.ac.kr/utr_designer/) (Seo et al., 2013b; 2013a). *egfp* ^*+*^ was expressed under these engineered HpdR UTRs. The sequences of the engineered UTRs of *hpdR* mRNA are shown in Supplementary Table S2. The engineered UTR fragments were PCR-amplified and cloned into pHRH_IA_eGFP^+^ at the *Xho*I/*Hind*III sites to generate plasmids with the engineered HpdR UTR, including pHRH^10f^_IA_eGFP^+^, pHRH^100f^_IA_eGFP^+^, pHRH^250f^_IA_eGFP^+^, pHRH^500f^_IA_eGFP^+^, pHRH^750f^_IA_eGFP^+^, and pHRH^1Kf^_IA_eGFP^+^. The engineered plasmids were individually introduced into electro-competent MG_*infA* ^−^ cells to generate the IRF^10f^, IRF^100f^, IRF^250f^, IRF^500f^, IRF^750f^, and IRF^1Kf^ strains. Additionally, eGFP^+^ in pHRH^750f^_IA_eGFP^+^ and pHRH^1Kf^_IA_eGFP^+^ was replaced with 3HBDH*/*Cb*FDH to construct pHRH^750f^_IA_3HBDH*/*Cb*FDH and pHRH^1Kf^_IA_3HBDH*/*Cb*FDH, respectively.

### Promoter assay

The efficiency of the constructed system was evaluated by analyzing the GFP fluorescence intensity of the recombinant strains with different LA concentrations. The recombinant strains were cultured overnight in LB medium and then subcultured (initial OD_600_ = 0.01) in 20 ml TB. When the OD_600_ reached ∼0.4, 180 µL of culture was aseptically inoculated into a clear-bottom Corning 96-well plate containing 5, 10, 20, 30, 40, 50, or 60 mM LA (20 µL). The LA stock (1 M) was prepared in TB and used to generate different concentrations of LA (5–60 mM). For the LacI/P_*LlacO1*_ system, 0.5 mM IPTG was added. The plate was incubated at 37°C with shaking. The GFP fluorescence intensity (gain of 30 at the excitation wavelength of 485 nm and emission wavelength of 535 nm) was then measured on a microplate fluorescence reader (Infinite F200 PRO, Tecan, Grődig, Austria). The fluorescence intensity was normalized based on the OD_600_ value of the culture. Subsequently, the culture was diluted appropriately with phosphate-buffered saline. Flow cytometry analysis of GFP fluorescence was performed using fluorescence-activated cell sorting (FACSCalibur Flow Cytometer, BD Bioscience; CA, United States). Approximately 2×10^5^ cells were analyzed per sample.

### 4-HV production by whole-cell biotransformation in a shake-flask

Whole-cell LA biotransformation was performed to produce 4-HV. Recombinant cells grown overnight in LB were inoculated into 500 ml TB to a final OD_600_ of 0.01. The strains were induced by adding 30 mM LA or 0.5 mM IPTG at 0.5–0.8 OD_600_. The strains were then cultured for at least 10 h. The recombinant cells were harvested by centrifugation at 12,000 × *g* for 15 min at 4°C, washed twice with potassium phosphate buffer (0.1 M, pH 6.0), and used as biocatalysts for biotransformation. The biotransformation was conducted in 100 ml shake flasks containing 20 ml potassium phosphate buffer (0.1 M, pH 6), an appropriate concentration of the induced resting cells, 23.2 g/L (0.2 M) LA, 13.6 g/L (0.2 M) sodium formate, and 1× filter sterilized trace element solution (g/L: 2.4 g FeCl_3_.6H_2_O, 0.3 g CoCl_2_·H_2_O, 0.15 g CuCl_2_.2H_2_O, 0.3 g ZnCl_2_, 0.3 g Na_2_MO_4_.2H_2_O, 0.075 g H_3_BO_3_, and 0.495 g MnCl_2_.4H_2_O) (Zhou et al., 2011; Kim D. et al., 2019). Sodium formate was used as the co-substrate. The biotransformations were conducted in a shaking incubator (37°C and 50 rpm), with sampling at different time points for product quantification by high-performance liquid chromatography (HPLC). To optimize the biocatalyst concentration, biotransformation was carried out with 10–60 g_wet cell weight (wcw)_/L cell. To ascertain plasmid stability, the RV (antibiotic-based plasmid maintenance) and IRV (*infA*-based plasmid maintenance) strains were serially subcultured for 10 days with 10 subcultures in the absence of Km. After the first, second, fourth, sixth, eighth, and 10th subcultures, the cells were prepared as described above and used for 4-HV production.

### Two-stage fed-batch 4HV production in a 5 L fermenter

Two-stage fed-batch 4HV production was carried out in a 5 L fermenter (MARADO-PDA; CNS, Daejeon, Korea) using IRV^750f^ harboring pHRH^750f^_IA_3HBDH*/*Cb*FDH under optimized conditions, as described previously (Kim D. et al., 2019). Briefly, in the first growth phase, the cells were grown to OD_600_ ≈ 50 with high aeration (0.5 vvm) and agitation (700 rpm) and induced by the addition of 30 mM LA at approximately 15 OD_600_. In the second biotransformation phase, a substrate solution containing LA and formate (final concentrations of 0.2 M each) was added at approximately 50–60 OD_600_. The agitation, aeration, and pH were maintained at 400 rpm, 0 vvm, and 6.0, respectively. A feeding solution containing 4 M LA (not neutralized), 4 M formate, and 0.4 M glycerol was fed in response to pH-stat (pH = 6). The feeding solution serving as a substrate and co-substrate source was automatically fed when the pH increased beyond 6. The samples were collected at different time points for product quantification by HPLC.

### Analytical methods

The cell density was monitored by measuring the absorbance at 600 nm (OD_600_) using a Biochrom Libra S22 spectrophotometer (Biochrom; Cambridge, United Kingdom). HPLC analysis [Shimadzu HPLC station (Shimadzu; Kyoto, Japan) equipped with a refractive index detector (Shimadzu) and a SIL-20A auto-sampler (Shimadzu)] was performed to quantify the levels of LA, 4-HV, formate, and glycerol in the cell-free supernatant from recombinant culture media. For this analysis, 500 µL of culture medium was collected and centrifuged at 16,000 × *g* for 20 min. The resulting supernatant was then heated at 80°C for 1 h to denature the remaining soluble proteins and centrifuged at 16,000 × *g* for 30 min to remove the denatured proteins. The final supernatant was then diluted 10-fold and analyzed by HPLC. To quantify the levels of LA and 4-HV, the samples were eluted through a 4.6 × 150 mm^2^, 5 μm Zorbax SB-Aq column (Agilent, United States) at 40°C using 25 mM ammonium formate (pH 2.0) as the mobile phase at a flow rate of 1 ml/min. To quantify the levels of glycerol and formate, the samples were eluted through a 300 × 7.8 mm^2^ Aminex HPX-87H column (Bio-Rad; United States) at 40°C using 5 mM H_2_SO_4_ as the mobile phase at a flow rate of 0.6 ml/min. The expression of GFP, 3HBDH*, and *Cb*FDH was evaluated using sodium dodecyl sulfate-polyacrylamide gel electrophoresis (SDS–PAGE). A culture sample (1 ml) was collected 12 h after induction, pelleted by centrifugation (16,000 × *g* for 1 min at 4°C), and washed twice with phosphate-buffered saline (pH 7.2). The cell pellet was then collected and resuspended in phosphate-buffered saline. The SDS-containing sample buffer was added to 15 µL of the sample, which was then heated for 10 min. A total of 12 µL of each sample was loaded onto the 10% acrylamide gel. The protein bands were visualized by staining with Coomassie Brilliant Blue R-250. The substrate conversion efficiency was calculated on a molar scale. All experimental data were subjected to one-way analysis of variance (ANOVA) or multivariate analysis of variance (MANOVA) using SPSS software (version 11, SPSS Inc.; Chicago, IL, United States) to determine the significance level. *p* < 0.05 was considered statistically significant.

## Results

### Evaluation of the LA-inducible HpdR/P_*hpdH*_ expression system

**FIGURE 2:**
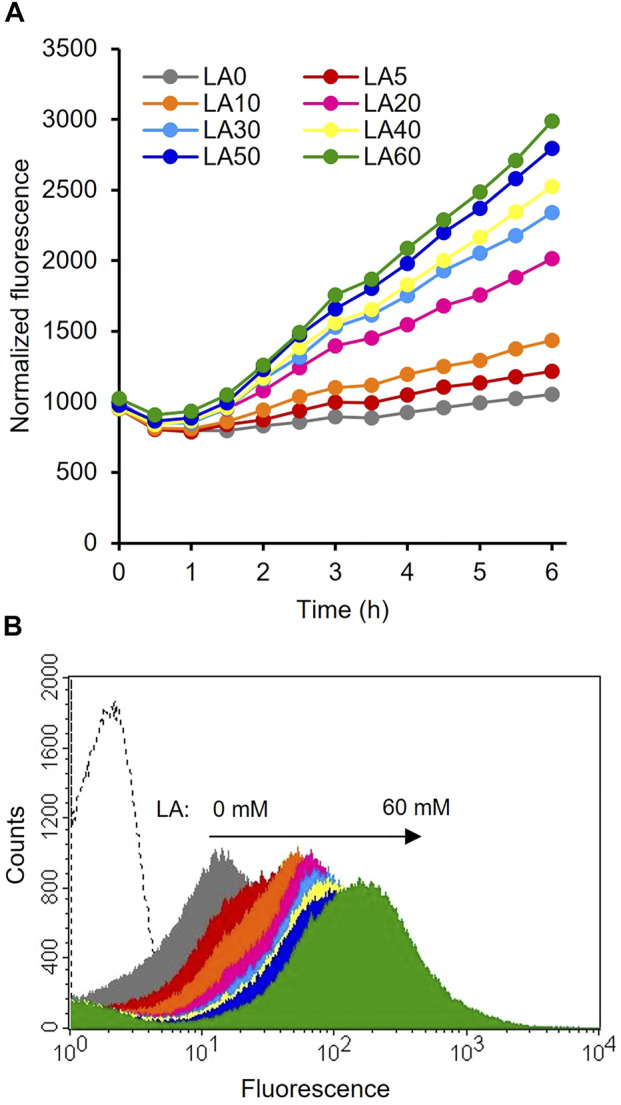
Evaluation of the LA-inducible HpdR/P_*hpdH*_ expression system in the IRF strain **(A)** Normalized LA-induced GFP fluorescence intensity of the HpdR/P_*hpdH*_ system. The strain, IRF (LA-inducible and *infA*-based plasmid maintenance), was induced by different LA concentrations (5–60 mM). Thus, the addition of LA increased the fluorescence intensity compared to that of the control (without LA). Data represent the mean fluorescence of three experiments. For clarity, error bars (standard deviation) are not shown in the figure **(B)** Flow cytometric analysis of the HpdR/P_*hpdH*_ system (strain IRF) induced by 30 mM LA. LA, levulinic acid.

The constructed plasmids pHRH_IA_eGFP^+^ and pLIL_IA_eGFP^+^ were individually transformed into MG_*infA* ^−^ to generate the IRF (*infA*-based plasmid maintenance; LA-inducible) and ILF (*infA*-based plasmid maintenance; IPTG-inducible) strains, respectively. The fluorescence intensities of these cultures were analyzed after cultivating the strains with their respective inducers. The results showed significantly increased fluorescence intensity of IRF (*p* < 0.05) after the addition of LA compared to that of the control (Figure 2A). This finding is consistent with those of previous reports on the LA-inducible HpdR/P_*hpdH*_ system in *P. putida* KT2440 (Sathesh-Prabu et al., 2021b) and *Methylorubrum extorquens* AM1 (Sathesh-Prabu et al., 2021a). Furthermore, the system was recognized to be tightly controlled (with no leaky expression). The fluorescence intensity increased as the LA concentration increased, up to 50 mM. However, the fluorescence intensity of IRF was 2.5-fold lower than that of 0.5 mM IPTG-induced ILF (Supplementary Figure S1). The GFP expression levels of the LA- and 3-HP-induced HpdR/P_*hpdH*_ systems did not differ significantly (Supplementary Figure S1). 3-HP is a native inducer of the HpdR/P_*hpdH*_ system (Zhou et al., 2015; Hanko et al., 2017; Sathesh-Prabu et al., 2021b). Figure 2B shows the flow cytometric results of IRF. IRF induced by different LA concentrations revealed tight control and the homogeneity (cell populations with similar expression levels) of the constructed expression system. Thus, the LA-inducible HpdR/P_*hpdH*_ system was used to produce 4-HV using 3HBDH* and *Cb*FDH.

### Evaluation and optimization of *infA*-based plasmid maintenance

**FIGURE 3:**
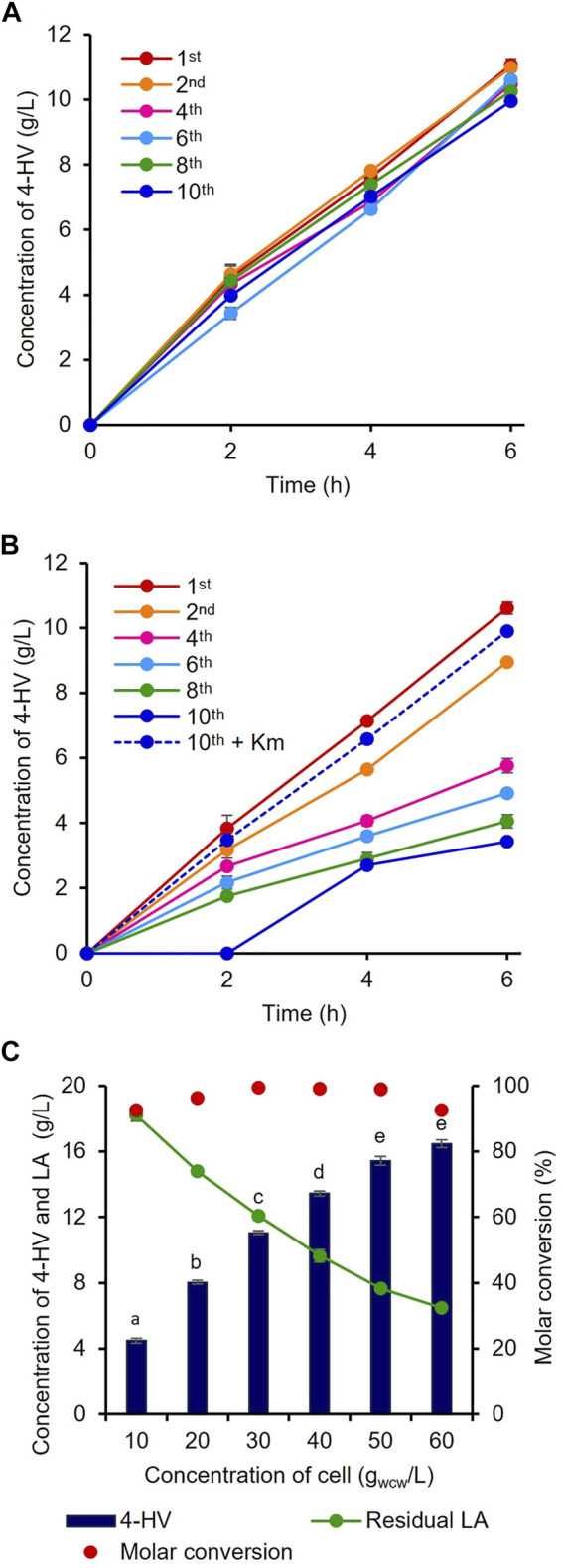
Evaluation of the antibiotic-free expression system. 4-HV produced by **(A)** IRV and **(B)** RV cells prepared after the first, second, fourth, sixth, eighth, and 10th subculture. IRV and RV containing *infA*-based and antibiotic-based plasmid for the expression of 3HBDH*/*Cb*FDH, respectively, subcultured without Km. Biotransformation was carried out with 30 g_wcw_/L recombinant cells. 4-HV production was reduced by a maximum of three-fold in RV after the 10th subculture. The dotted line in Figure 3B indicates the RV strain subcultured 10 times with Km **(C)** Optimization of the cell concentration. Different concentrations of cell biomass (10–60 g_wcw_/L) of IRV. Approximately 23 g/L of LA was added for the biotransformation. Beyond 50 g_wcw_/L cells, there was no significant increase in product titer (*p* > 0.05). 4-HV was measured at 6 h after biotransformation. Data represent the mean titer of three experiments, and error bars represent standard deviation. Different letters above the bars indicate statistically significant difference at *p* < 0.05.4-HV, 4-hydroxyvaleric acid; Km, kanamycin.

The constructed plasmids pHRH_IA_3HBDH*/*Cb*FDH and pHRH_3HBDH*/*Cb*FDH were individually transformed into MG_*infA* ^−^ and WT to generate the *infA*-based plasmid-maintaining strain IRV (LA-inducible) and antibiotic-based plasmid-maintaining strain RV (LA-inducible), respectively, for 4-HV production by whole-cell LA biotransformation. To analyze the efficiency of the constructed antibiotic-free 4-HV production system, IRV and RV were cultured and continuously subcultured (1:100) in fresh medium without Km at least 10 times. During passaging, 30 g_wcw_/L cells were prepared after the first, second, fourth, sixth, eighth, and 10th subcultures and used to produce 4-HV from LA. Notably, the IRV efficiency was not significantly affected by the absence of Km (*p* < 0.05) even after 10 subcultures (Figure 3A). The IRV produced similar amounts of 4-HV after the first (11 g/L) and 10th (10 g/L) subcultures. In contrast, the production efficiency (three-fold lesser titer) of RV was significantly affected in the absence of Km (Figure 3B). 4-HV production gradually decreased (1.19- to 3.08-fold) as the number of subcultures increased. The production rate also decreased as the number of subcultures increased. After the 10th subculture, no 4-HV was produced by RV, even 2 h after biotransformation. However, the amounts of 4-HV produced by RV subcultured (10 times) with Km and the cells of the first subculture were similar (Figure 3B). Only 45% of RV cells grew on LB agar supplemented with Km when a 10th subculture aliquot was plated on LB agar with and without Km. These findings suggested that 55% of RV cells had lost their plasmid (Supplementary Figure S2), whereas 100% of IRV cells in the 10th subculture grew on LB agar supplemented with Km.

### 4-HV production by whole-cell biotransformation at the shake-flask level

**FIGURE 4:**
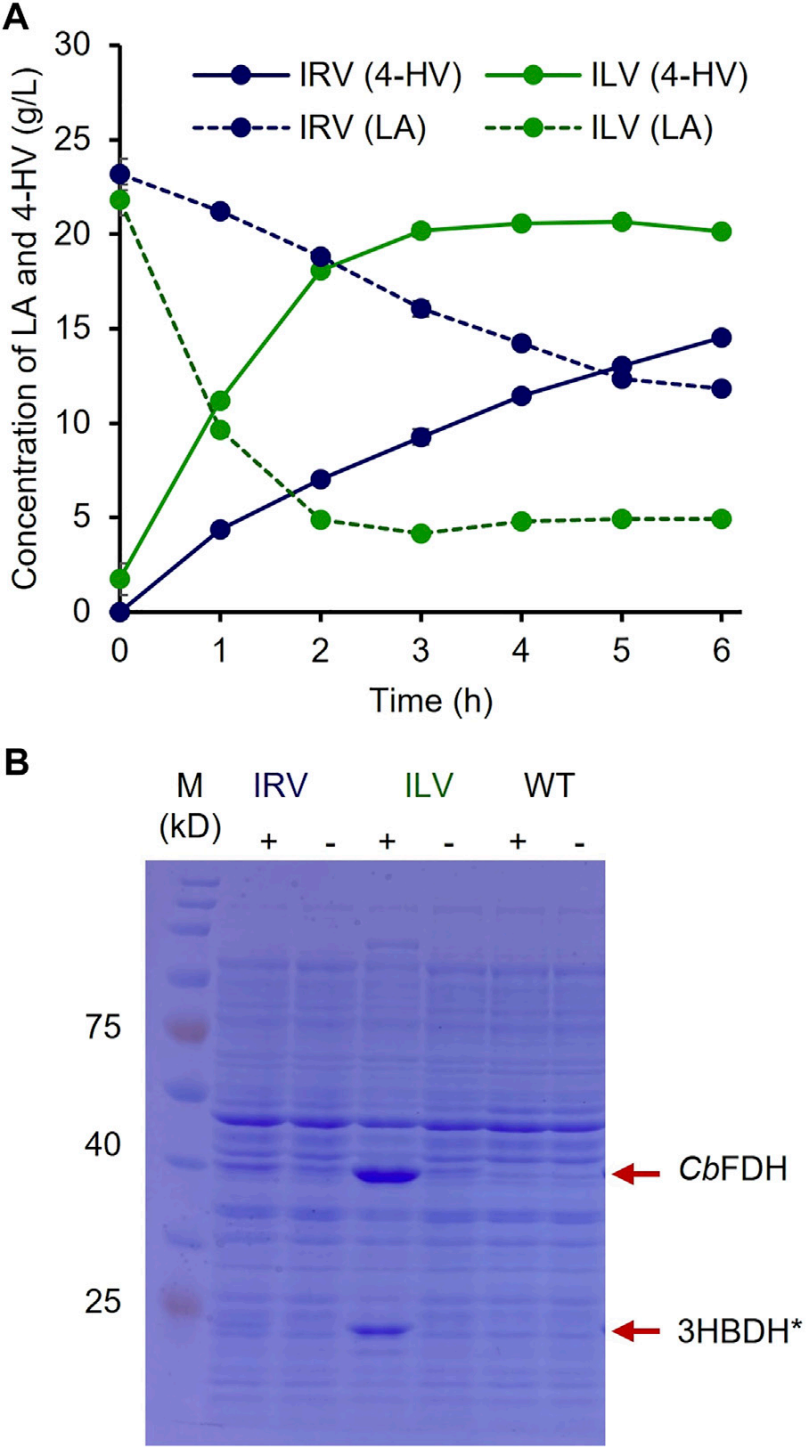
4-HV production by IRV and ILV under optimized conditions **(A)** 4-HV produced by IRV (LA-inducible) and ILV (IPTG-inducible) under optimized conditions (50 g_wcw_/L, 37°C, and 50 rpm) and analyzed during 6 h of biotransformation at 1 h intervals. The strains, IRV and ILV, produced approximately 15 and 20 g/L of 4-HV after 6 and 3 h of biotransformation, respectively. Solid and dotted lines indicate the produced 4-HV and residual LA, respectively. Data represent the mean of three experiments, and error bars represent standard deviation **(B)** SDS–PAGE analysis of the recombinant cells, IRV and ILV. The strains were induced by adding 30 mM LA or 0.5 mM IPTG. The expression levels of 3HBDH* and *Cb*FDH (indicated by red arrows) in IRV were meager compared to those in ILV. 3HBDH*, engineered 3-hydroxybutyrate dehydrogenase; 4-HV, 4-hydroxyvaleric acid; *Cb*FDH, formate dehydrogenase; IPTG, isopropyl β-D-1- thiogalactopyranoside; LA, levulinic acid; SDS–PAGE, sodium dodecyl sulfate polyacrylamide gel electrophoresis; +, w/ inducer; -, w/o inducer.

To optimize the biocatalyst concentration for maximum 4-HV production, biotransformation was performed with 10, 20, 30, 40, 50, and 60 g_wcw_/L of IRV. The product titer was significantly influenced by cell concentration (*p* < 0.05). Although the product titers were within 4–16 g/L, the molar conversion (%) was not considerably affected (93–99%) under all conditions. 4-HV produced by 50 g_wcw_/L was increased by a maximum of 3.5-fold from that produced by 10 g_wcw_/L (Figure 3C). Beyond 50 g_wcw_/L cells, the product titer did not increase significantly (*p* > 0.05). Therefore, 50 g_wcw_/L cells were used in further studies. IRV was used for 4-HV production under optimized conditions (50 g_wcw_/L, 37°C, pH 6.0, and 50 rpm), and its efficiency was compared to that of ILV. IRV produced ∼15 g/L of 4-HV 6 h after biotransformation, demonstrating the functional expression of the constructed system (Figure 4A). The titer gradually increased with biotransformation duration but did not increase 6 h after biotransformation (data not shown). In contrast, the maximum amount of 4-HV produced by ILV (approximately 20 g/L) occurred 3 h after biotransformation, demonstrating a 2.8-fold higher productivity rate than that of IRV (Figure 4A). SDS-PAGE analysis of IRV and ILV showed lower expression levels of 3HBDH* and *Cb*FDH in IRV than those in ILV (Figure 4B). The slow production rate in IRV might have been due to insufficient 3HBDH* and *Cb*FDH expression. Therefore, increasing the expression level of the HpdR (transcriptional activator) regulatory protein in the LA-inducible system could, in turn, increase 3HBDH* and *Cb*FDH expression in a controlled manner.

### Evaluation of the engineered HpdR/P_*hpdH*_ system

**FIGURE 5:**
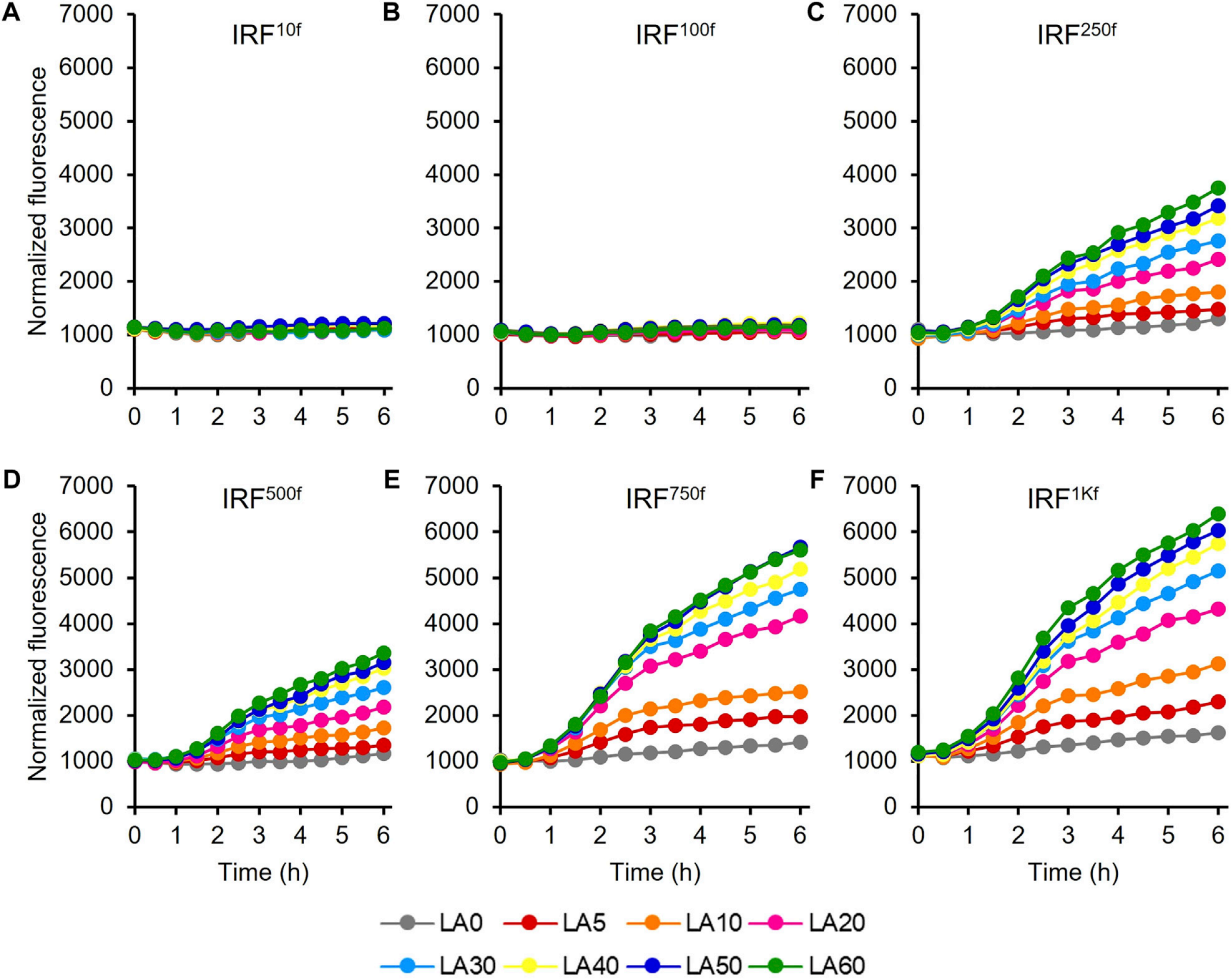
Evaluation of the 5′ UTR engineered HpdR/P_*hpdH*_ expression systems in the IRF strain. Normalized GFP fluorescence intensity of the 5′ UTR engineered HpdR/P_*hpdH*_ expression systems. The UTR of *hpdR* mRNA in pHRH_IA_eGFP^+^ was engineered. The fluorescence intensity of the recombinant strains **(A)** IRF^10f^, **(B)** IRF^100f^, **(C)** IRF^250f^, **(D)** IRF^500f^, **(E)** IRF^750f^, and **(F)** IRF^1Kf^ exhibiting 10-, 100-, 250-, 500-, 750-, and 1000-fold stronger HpdR expression than the native system, respectively, was evaluated using 5–60 mM LA. The expression level increased when the strength of the HpdR UTR increased beyond 100-fold relative to that of the native system. Data represent the mean of three experiments. For clarity, error bars (standard deviation) are not shown in the figures. GFP, green fluorescent protein; UTR, untranslated region.

**FIGURE 6:**
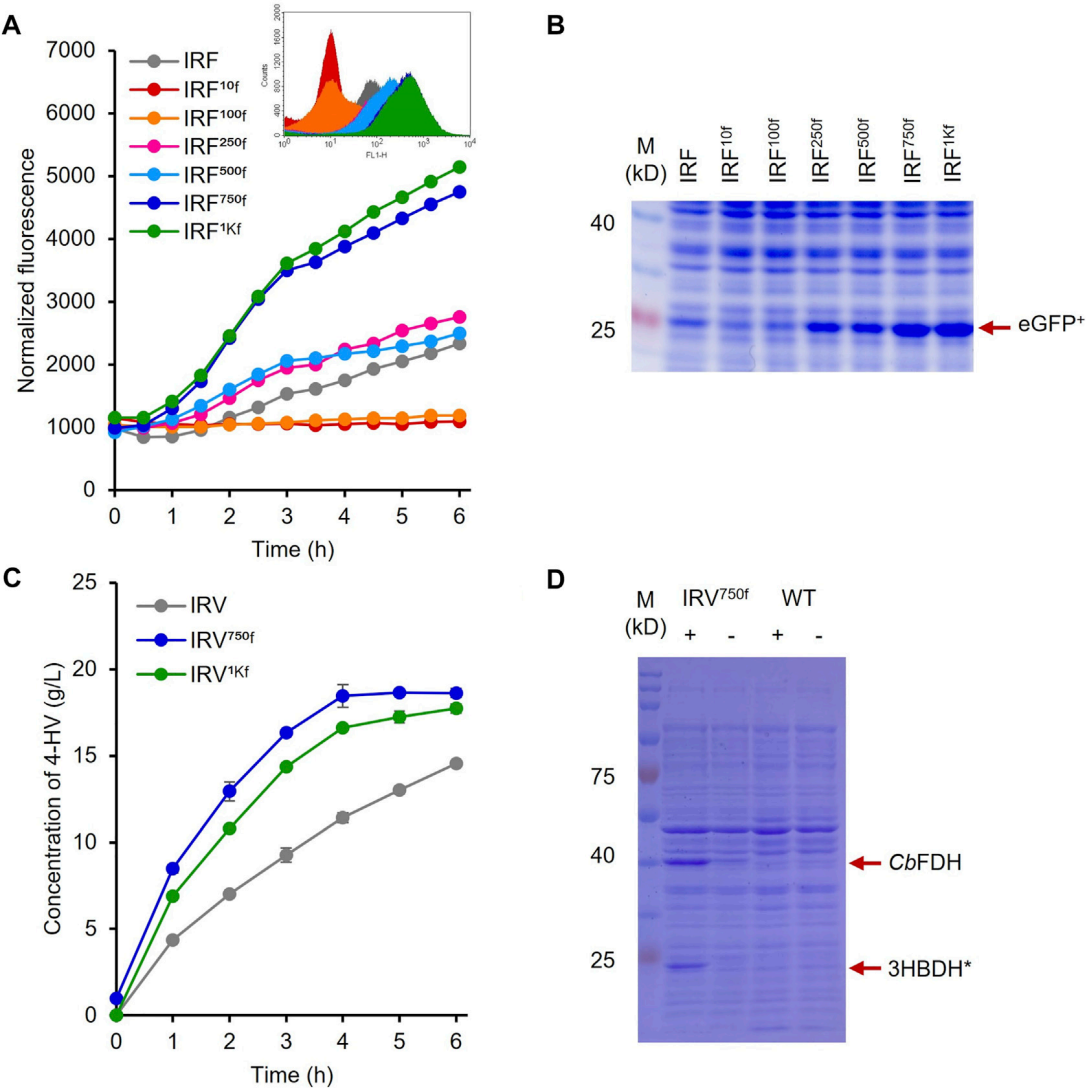
Protein and 4-HV production by the 5′ UTR engineered HpdR/P_*hpdH*_ expression systems **(A)** Comparison of the normalized GFP fluorescence intensity of the recombinant strains IRF^10f^, IRF^100f^, IRF^250f^, IRF^500f^, IRF^750f^, and IRF^1Kf^ with that of the native system, IRF induced by 30 mM LA. IRF^750f^ showed at least a two-fold increase in fluorescence intensity relative to IRF. Inset: Flowcytometric analysis of the recombinant strains **(B)** SDS–PAGE analysis of the 5′ UTR engineered strains induced by 30 mM LA. The expression of eGFP^+^ (indicated by red arrow) increased in response to increasing UTR strength **(C)** 4-HV production by IRV^750f^ and IRV^1Kf^. 4-HV production by IRV^750f^ was approximately 77.5% higher than that by the native system, IRV **(D)** SDS–PAGE analysis of IRV^750f^. The expression levels of 3HBDH* and *Cb*FDH (indicated by red arrow) by IRV^750f^ were higher than those by the native system, IRV. Data represent the mean of three experiments, and error bars represent standard deviation. 3HBDH*, engineered 3-hydroxybutyrate dehydrogenase; 4-HV, 4-hydroxyvaleric acid; *Cb*FDH, formate dehydrogenase; GFP, green fluorescent protein; LA, levulinic acid; SDS–PAGE, sodium dodecyl sulfate polyacrylamide gel electrophoresis; UTR, untranslated region.

The UTR of the *hpdR* mRNA in pHRH_IA_eGFP^+^ was engineered by replacing its native ribosome-binding site (RBS) with a rationally designed RBS to modulate HpdR expression. The fluorescence intensities of the recombinant strains IRF^10f^, IRF^100f^, IRF^250f^, IRF^500f^, IRF^750f^, and IRF^1Kf^ exhibiting 10-, 100-, 250-, 500-, 750-, and 1000-fold higher levels of HpdR expression relative to the native system, respectively, were evaluated using 5–60 mM LA. Although the UTR strength in IRF^100f^ increased up to 100-fold relative to that in the native system, the *egfp* ^+^ expression in IRF^100f^ was lower than that in the native system (Figure 5; Figure 2A). However, the expression level increased when the HpdR UTR strength increased beyond 100-fold relative to that in the native system. The expression levels were controlled and regulated using different LA concentrations. IRF^750f^ showed at least a two-fold increase in fluorescence intensity relative to the native system (Figure 5; Figure 6A). Moreover, the IRF^750f^ efficiency was 75% that of the ILF (IPTG-inducible) efficiency. Herein, expression was controlled by varying the concentration of LA. The *egfp* ^+^ expression levels did not differ significantly between IRF^750f^ and IRF^1Kf^. However, the expression level may be saturated in IRF^750f^. Regulatory protein expression is crucial for an inducible expression system to obtain high levels of protein expression. Flow cytometric analysis of the UTR engineered HpdR/P_*hpdH*_ systems revealed tight control and the homogeneity of the engineered expression systems (Supplementary Figure S3). The *egfp* ^+^ expression in the 30 mM LA-induced recombinant strains analyzed by SDS-PAGE showed increasing protein band intensity with increasing HpdR RBS strength (Figure 6B).

As the eGFP^+^ expression levels were increased in IRF^750f^ and IRF^1Kf^, the HpdR UTRs of these strains were used to construct IRV^750f^ and IRV^1kf^ harboring the plasmids, pHRH^750f^_IA_3HBDH*/*Cb*FDH and pHRH^1Kf^_IA_3HBDH*/*Cb*FDH, respectively, which produced a maximum of 19 g/L of 4-HV at 6 h after biotransformation. More than 80% of the 4-HV was produced within 3 h of biotransformation. The productivities of IRV, IRV^750f^, and IRV^1Kf^ were 3.1, 5.5, and 4.8 g/L/h, respectively. Thus, IRV^750f^ displayed ∼76% higher efficiency compared to IRV (Figure 6C). Moreover, the production rates induced by IRV^750f^ and IRV were 81 and 46% of those induced by ILV (Figure 6C; Figure 4A). Figure 6D shows the expression of 3HBDH* and *Cb*FDH in IRV^750f^, as assessed by SDS-PAGE. This increased level of protein expression was reflected in the high production rate induced by IRV^750f^. Therefore, IRV^750f^ was used for fed-batch production of 4-HV in a 5 L fermenter.

### Two-stage fed-batch production of 4-HV in a 5 L fermenter

**FIGURE 7:**
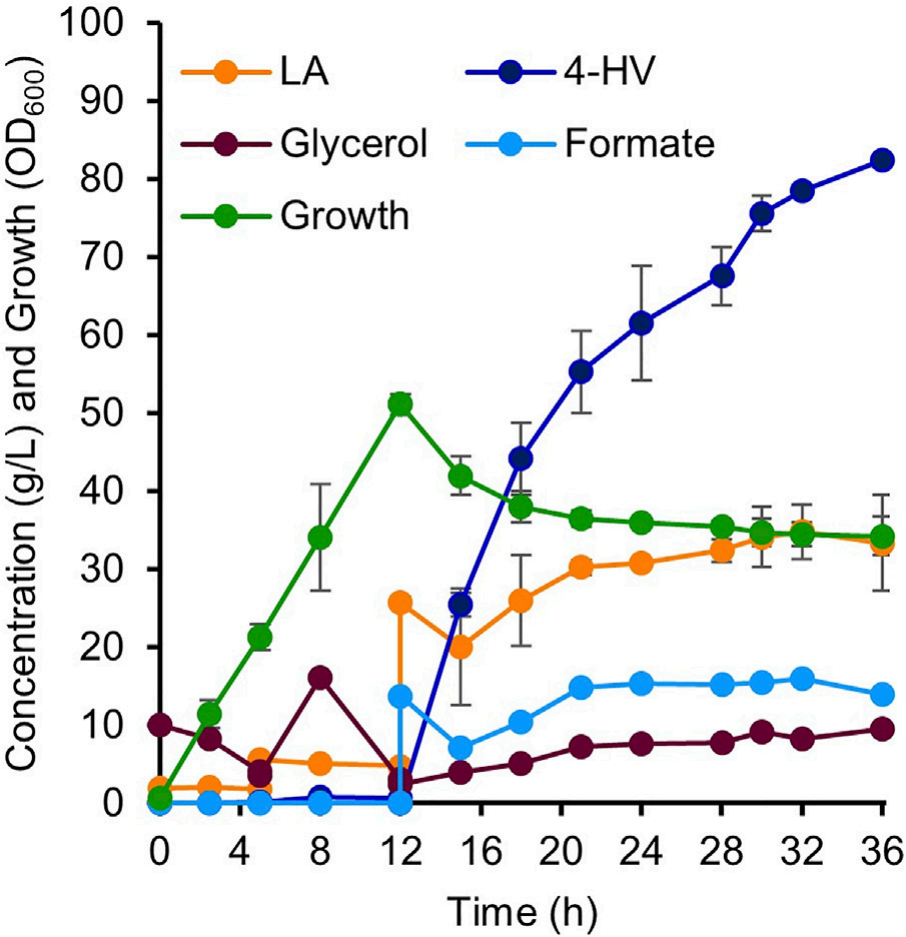
4-HV production by two-stage fed-batch cultivation in a 5 L fermenter. Detailed parameters of 4-HV biosynthesis by IRV^750f^. Biotransformation was initiated by the addition of LA and formate (final concentration: 0.2 M each). A maximum of 82 g/L of 4-HV with a molar conversion of 92% was achieved after 24 h of biotransformation. Data represent the mean of three independent experiments and error bars represent standard deviation. 4-HV, 4-hydroxyvaleric acid; LA, levulinic acid.

Two-stage (growth and production phases) fed-batch production of 4-HV was performed using IRV^750f^ in a 5 L fermenter. During the growth phase, the culture reached an OD_600_ of 50 after 12 h of inoculation. LA and formate were then added to initiate the production phase. Figure 7 shows various parameters, including LA, glycerol, and formate concentrations; 4-HV production; and IRV^750f^ growth during 4-HV biosynthesis under the optimized conditions. IRV^750f^ produced a maximum of 82 g/L of 4-HV at 24 h after substrate addition. Approximately 76% of 4-HV (62 g/L) was produced within 12 h of substrate addition, corresponding to a productivity of 5.2 g/L/h. Collectively, IRV^750f^ produced 82 g/L of 4-HV, corresponding to a productivity of 3.4 g/L/h and a 92% molar conversion at 24 h after substrate addition, which is ∼18% less than that produced by the IPTG-inducible system. Therefore, the system presented in this study may be useful for biochemical production, particularly in LA biorefineries, even at a large scale, without requiring the use of antibiotics or expensive inducer chemicals such as IPTG.

## Discussion

In this study, an engineered *E. coli* strain produced ∼ 82 g/L of 4-HV without the addition of antibiotics or costly inducers. 4-HV is an industrially relevant monomer of biodegradable polymers and a precursor of γ-valerolactone, which is used as a fuel, solvent, food additive, and precursor for value-added carbon chemicals (Gorenflo et al., 2001; Bond et al., 2010; Yan et al., 2015). In addition to the chemical synthesis of 4-HV, 50–100 g/L of 4-HV has been biologically produced using novel pathways and engineered microbial strains (Kim D. et al., 2019; Sathesh-Prabu and Lee, 2019; Moon et al., 2021). Notably, these production systems require dedicated plasmids, antibiotics, and expensive inducers to generate multiple gene copies, select and retain the plasmid, and efficiently induce the expression of synthetic pathway genes.

The addition of antibiotics and inducers is associated with limitations including (i) their cost; (ii) labor-intensive nature; (iii) the additional stress on the cell; (iv) the gradual degradation of antibiotics and inducers; and (v) the dissemination of antibiotic resistance that should be addressed for long-term cultivation or large-scale production of biochemicals and proteins. The use of antibiotics can be avoided by integrating synthetic pathway(s) into the chromosome (Saleski et al., 2021) or creating antibiotic-free plasmid maintenance systems (Mignon et al., 2015; Kang C. W. et al., 2018). However, integrating synthetic pathway(s) into the chromosome has some limitations, such as a low dosage of target genes, leading to inefficient biochemical production, and the influence of the chromosomal position used for integration on gene expression (Bryant et al., 2014). Several methods have been reported for creating antibiotic-free plasmid maintenance systems (Mignon et al., 2015). For instance, the deletion of genes involved in the biosynthesis of amino acids or nicotinamide adenine dinucleotide and the subsequent complementation of those deleted genes by the plasmids have been reported for the construction of antibiotic-free plasmid maintenance systems (Vandermeulen et al., 2011). However, these auxotrophic mutants can survive in a common rich medium, such as LB, owing to the presence of amino acid or nicotinamide adenine dinucleotide precursors. Complementing essential genes such as *infA* is a feasible strategy due to the lack of toxicity, off-target effects, and cross-contamination, in addition to the tight control (Kang C. W. et al., 2018). Another main advantage of using *infA* is its protein size (72 amino acids), which allows the construction and maintenance of a small plasmid without inducing an additional burden on the host cell. *infA* is a highly conserved element of the bacterial translational apparatus and is essential for cell viability (Cummings and Hershey, 1994). *infA* is involved in the initiation phase of protein synthesis by stabilizing the binding of initiation factors 2 and 3 on the 30S subunit, where the binding of initiator tRNA and new mRNA occurs, yielding the 30S pre-initiation complex (Cummings and Hershey, 1994). Therefore, in this study, *infA* was deleted from the WT and expressed by the plasmid along with the 4-HV synthetic pathway genes. Thus, the strain (*infA*-based plasmid maintenance system) was dependent on the *infA*-harboring plasmid for growth. The 4-HV titer was reduced by three-fold in the RV strain harboring the antibiotic-based plasmid, pHRH_3HBDH*/*Cb*FDH, after 10 subcultures without antibiotics, corresponding to a 55% plasmid loss. The *infA*-based plasmid (IRV; pHRH_IA_3HBDH*/*Cb*FDH) did not significantly (*p* < 0.05) influence 4-HV production efficiency even after 10 subcultures without antibiotics (Figure 3), demonstrating the robustness and plasmid stability of the constructed system. This finding is consistent with the stable maintenance of the *infA*-based plasmid for 40 generations with minimized cell-to-cell variation recently reported by Kang C. W. et al. (2018). Therefore, this strategy could be effectively used to produce biochemicals under antibiotic-free conditions.

The present study used an LA-inducible HpdR/P_*hpdH*_ expression system to avoid the use of expensive chemical inducers such as IPTG. To our knowledge, this is the first report of an LA-inducible HpdR/P_*hpdH*_ expression system in *E. coli*. The system efficiently induced eGFP^+^ expression in cells via the addition of LA and tightly controlled and regulated expression in a dose-dependent manner. This approach is advantageous in synthetic biology and metabolic engineering (basic and applied research) as it can be used to optimize metabolic pathways to achieve optimum gene expression, high product titers, yields, and productivity (Jones et al., 2015). We confirmed the robustness of our system through the production of the industrially-relevant chemical, 4-HV. However, an analysis of eGFP^+^ expression in *E. coli* showed that the sensitivity and efficiency of the LA-inducible HpdR/P_*hpdH*_ system were at least six-fold lower than those in *P. putida* KT2440 (Sathesh-Prabu et al., 2021b), which may be attributed to the reduced heterologous HpdR activity in *E. coli*. However, a two-fold increased expression of eGFP^+^ was achieved even with 20 mM of LA relative to the control condition (0 mM). The inducibility of LA has several benefits for biorefineries, as renewable LA serves as a starting material for several fine and bulk chemicals, with applications in polymers, plasticizers, fuels, resins, fragrances, pharmaceuticals, anti-freeze agents, and solvents (Bozell et al., 2000; Rackemann and Doherty, 2011; Pileidis and Titirici, 2016; Sathesh-Prabu and Lee, 2019; Cha et al., 2020; Hayes and Becer, 2020). LA reduces production costs and eases induction and the downstream processes. As a substrate/inducer, LA can be readily obtained as the major product of the variously hydrolyzed renewable cellulosic biomass, hexose (the theoretical yield of LA from hexose is 64.4 wt%) (Kang S. et al., 2018). In addition, the co-substrate, formate, is produced during the conversion of cellulosic hexose sugars to LA. Equimolar production of LA and formate was obtained from cellulosic biomass such as corn cob and rice straw (Moon et al., 2021). Therefore, the presented system could be useful to produce LA-derived chemicals under the LA-inducible expression system.

During the optimization of biocatalyst concentration, cell concentration was shown to affect the product titer but not the substrate conversion efficiency (Figure 3A). This finding reflects efficient LA conversion (the substrate was not degraded by the host cell) and insufficient 3HBDH* and *Cb*FDH protein expression/function for the maximum conversion of LA into 4-HV. This result was also demonstrated via SDS-PAGE analysis, as the bands for 3HBDH* and *Cb*FDH were not prominent (Figure 4B). Furthermore, the productivity rate of the IPTG-inducible system (ILV) was 2.8-fold higher than that of IRV (Figure 3A). HpdR is a transcriptional activator involved in 3-HP catabolism in *Pseudomonas* spp. (Zhou et al., 2015; Hanko et al., 2017). Upon binding of the effector molecules, such as 3-HP or LA, to HpdR, the resulting conformational changes in the activator enhance RNA polymerase binding in the promoter sequence of the downstream genes, ultimately allowing genes transcription (Zhou et al., 2015; Oliver et al., 2016; Hanko et al., 2017; Sathesh-Prabu et al., 2021b). Low HpdR expression may cause the meager 3HBDH*/*Cb*FDH expression, as sufficient expression of a transcriptional regulator is essential for the regulation of protein expression. As expected, the expression system with engineered *hpdR* mRNA UTR increased eGFP^+^ expression. Moreover, the protein bands were prominent compared to those of the native system (Figure 6). The production rate of 4-HV increased from 3.1 to 5.5 g/L/h by IRV^750f^. In addition, the UTR engineered systems could be fine-tuned time- and dose-dependently, which is helpful for precise control of gene expression (Garcia-Perez et al., 2022). The productivity of IRV^1Kf^ was lower than that of IRV^750f^ (4.8 vs. 5.5 g/L/h). When overexpressed, a transcription factor can act as a repressor or behave aberrantly (Lee et al., 1998). Therefore, the precise control of transcription regulator expression is essential for the maximum expression of target genes to achieve good performance. Kim D. et al. (2019) reported an 82% efficiency of the IRV^750f^ strain relative to the highest 4-HV producing strain (100 g/L of 4-HV) in fed-batch cultivation, using an antibiotic-based plasmid and IPTG-inducible system.

An uncontrolled high-level expression or constitutive expression system is not always desirable for biochemical production and the use of antibiotics and IPTG is not suitable for industrial scale-up. Therefore, the use of inexpensive renewable substrates as inducers and antibiotic-free plasmid maintenance could be a feasible strategy for large-scale biochemical production. This study engineered and evaluated a renewable LA-inducible and tunable gene expression system, HpdR/P_*hpdH*_, for use in *E. coli*. We demonstrated the usefulness of this engineered system for establishing sustainable and economic biochemical production in antibiotic- and chemical inducer-free conditions in *E. coli*, a widely used biotechnological host.

## Data Availability

The original contributions presented in the study are included in the article/Supplementary Material. Further inquiries can be directed to the corresponding author.
