# Supplementary material for: Substrate-inducible and antibiotic-free high-level 4-hydroxyvaleric acid production in engineered Escherichia coli
Source: Front Bioeng Biotechnol. 2022 Aug 9;10:960907. doi: 10.3389/fbioe.2022.960907 (PMC9398171; doi:10.3389/fbioe.2022.960907)
Supplement: Supplementary file 1 [file DataSheet1.PDF]

Table S1. Oligomers used in the study

Primers	Sequences (5’ → 3’)	Purpose
InfA_Gib_FP<br>InfA_Gib_RP	ggcttacccgtcttactgtccacaaaataaaaaggccg<br>atccactgaaagcacagcgggaatgaatgttttcggcac	To amplify *infA* from genomic DNA of *E. coli* MG1655. The amplicon was used to construct pSIM5_*infA*.
InfA_pPBE_FP<br>InfA_pPBE_RP	gaattggggatcggaagctgaattccacaaaataaaaaggccg<br>actgagcctttcgttttatgaattcgaatgaatgttttcggcac	To amplify *infA* from genomic DNA of *E. coli* MG1655. The amplicon was used to construct pHRH_IA_eGFP^{+}.
3HBDH*_FP<br>3HBDH*_RP	ctttataaggaggaaaaacatatgcttaaaggtaaaaaagcagtc<br>agtccaagctcagctaattaagcttagatccgacgtctcatcttgcggtccaacc	To amplify 3HBDH* from pBbE6k_3HBDH* to construct pHRH_IA_3HBDH*.
*Cb*FDH_FP<br>*Cb*FDH_RP	tggaccgcaagatgagacgtctttaagaaggagatatacacatgaaaattgttc tggtgc<br>agtccaagctcagctaattaagcttttattttttatcgtgtttaccg	To amplify CbFDH from pBbB6a_*Cb*FDH and to fuse it with 3HBDH* to construct pHRH_IA _3HBDH*/*Cb*FDH.
LacI/P$_{LlacO1}$_FP<br>LacI/P$_{LlacO1}$_RP	ggcatttcgttttatgagctctcactgcccgctttccag<br>ttaagcggctgcagtcgacggatccatgtatatctccttcttaaa agatcttttgaattcg	To amplify LacI/ P$_{LlacO1}$ from pBbE6k_RFP and clone into pHRH_IA _3HBDH*/*Cb*FDH to pLIL_IA_3HBDH*/*Cb*FDH.
eGFP^{+}_FP<br>eGFP^{+}_RP	ctttataaggaggaaaaacatatgagtaaaggagaagaac<br>agtccaagctcagctaattaagcttatttgtagagctcatcc	To amplify eGFP^{+} from pPROBE_eGFP^{+} and clone into pLIL_IA _3HBDH*/*Cb*FDH to construct pLIL_IA_eGFP^{+}.
3HBDH*/*Cb*FDH_FP<br>3HBDH*/*Cb*FDH_RP	ctttataaggaggaaaaacatatgcttaaaggtaaaaaagc<br>agtccaagctcagctaattaagcttttattttttatcgtgtttaccg	To amplify 3HBDH/FDH from pHRH_IA_3HBDH*/*Cb*FDH and clone into pHRH_eGFP^{+}, pHRH^{750f}_IA_eGFP^{+}, and pHRH^{1000f}_IA_eGFP^{+} to construct pHRH_3HBDH*/*Cb*FDH, pHRH^{750f}_IA_3HBDH*/*Cb*FDH and pHRH^{1000f}_IA_3HBDH*/*Cb*FDH, respectively.
10fUTR_eGFP^{+}_FP<br>xF_ eGFP^{+}_RP	agcggccgctgcgctgcaactcgaggaaaaaccgcagatcattccagtcgaacatgct gaagccgtctggcagttcgttccggcc<br>agtccaagctcagctaattaagcttatttgtagagctcatccatgccatgtg	To amplify 10f UTR and eGFP^{+} from pHRH_eGFP^{+} to construct pHRH^{10f}_IA_eGFP^{+}.
100fUTR_eGFP^{+}_FP	agcggccgctgcgctgcaactcgaggaaaaaccgcagatcattccagtcgaacatgct acccaccgatggcagttcgttccggcc	To amplify 100f UTR and eGFP^{+} from pHRH_eGFP^{+} to construct pHRH^{100f}_IA_eGFP^{+} with xF_ eGFP^{+}_RP.
250fUTR_eGFP^{+}_FP	agcggccgctgcgctgcaactcgaggaaaaaccgcagatcattccagtcgaacatgct gtgtactcctggcagttcgttccggcc	To amplify 250f UTR and eGFP^{+} from pHRH_eGFP^{+} to construct pHRH^{250f}_IA_eGFP^{+} with xF_ eGFP^{+}_RP.
500fUTR_eGFP^{+}_FP	agcggccgctgcgctgcaactcgaggaaaaaccgcagatcattccagtcgaacatgct tgtccaccttggcagttcgttccggcc	To amplify 500f UTR and eGFP^{+} from pHRH_eGFP^{+} to construct pHRH^{500f}_IA_eGFP^{+} with xF_ eGFP^{+}_RP.
750fUTR_eGFP^{+}_FP	agcggccgctgcgctgcaactcgaggaaaaaccgcagatcattccagtcgaacatgct tgaactccttggcagttcgttccggcc	To amplify 750f UTR and eGFP^{+} from pHRH_eGFP^{+} to construct pHRH^{750f}_IA_eGFP^{+} with xF_ eGFP^{+}_RP.
1000fUTR_eGFP^{+}_FP	agcggccgctgcgctgcaactcgaggaaaaaccgcagatcattccagtcgaacatgct aacctcccgtggcagttcgttccggcc	To amplify 1000f UTR and eGFP^{+} from pHRH_eGFP^{+} to construct pHRH^{1000f}_IA_eGFP^{+} with xF_ eGFP^{+}_RP.

Table S2. Sequences of the engineered UTRs of *hpdR* mRNA

UTR variants	5'-UTR Sequence of HpdR	Fold higher than native system
Native	GAACGAACTGCCATAAAGGACAAGC	1
10f	GAACGAACTGCCAGACGGCTTCAGC	10
100f	GAACGAACTGCCATCGGTGGGTAGC	100
250f	GAACGAACTGCCAGGAGTACACAGC	250
500f	GAACGAACTGCCAAGGTGGACAAGC	500
750f	GAACGAACTGCCAAGGAGTTCAAGC	750
1000f	GAACGAACTGCCACGGGAGGTTAGC	1000

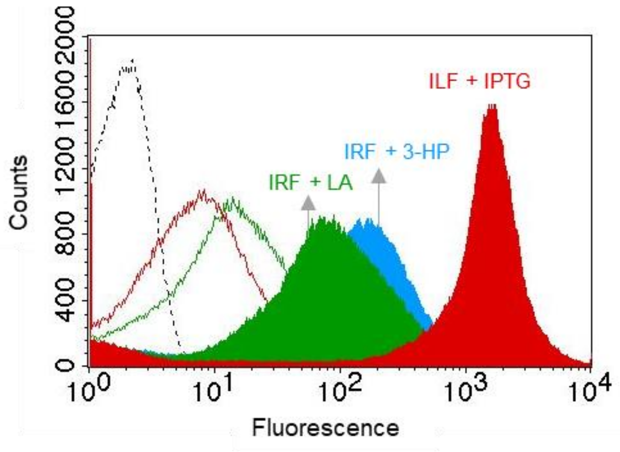

Supplementary Figure S1: **Flow cytometric analysis of IRF and ILF**. IRF was induced with 30 mM of LA or 30 mM of 3-HP. ILF was induced with 0.5 mM of IPTG. Filled peaks represent the fluorescence of the strains induced by corresponding inducers, whereas the solid line peak (green and red) indicates the fluorescence of the strains without inducers. Dotted peak (black) refers to the WT.

IRV

LB + Km

A

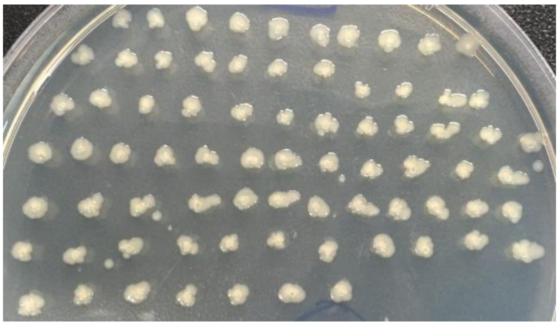

Plasmid lost: 0 %

RV

LB + Km

B

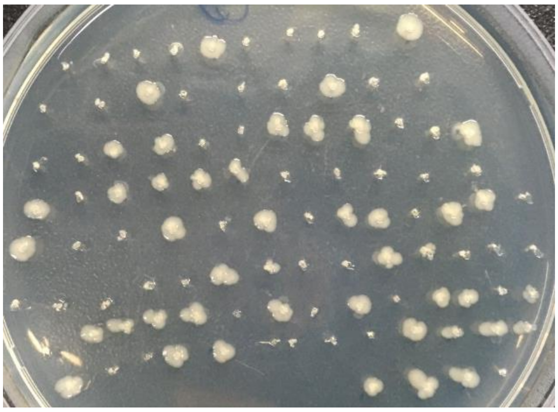

Plasmid lost: 55 %

Supplementary Figure S2: **Analysis of plasmid stability**. An aliquot of tenth subculture (cultivated without Km) of the strains IRV (*infA*-based) and RV (Km-based) was spread on an LB agar plate without Km. Subsequently, randomly selected colonies were inoculated onto an LB agar supplemented with Km.

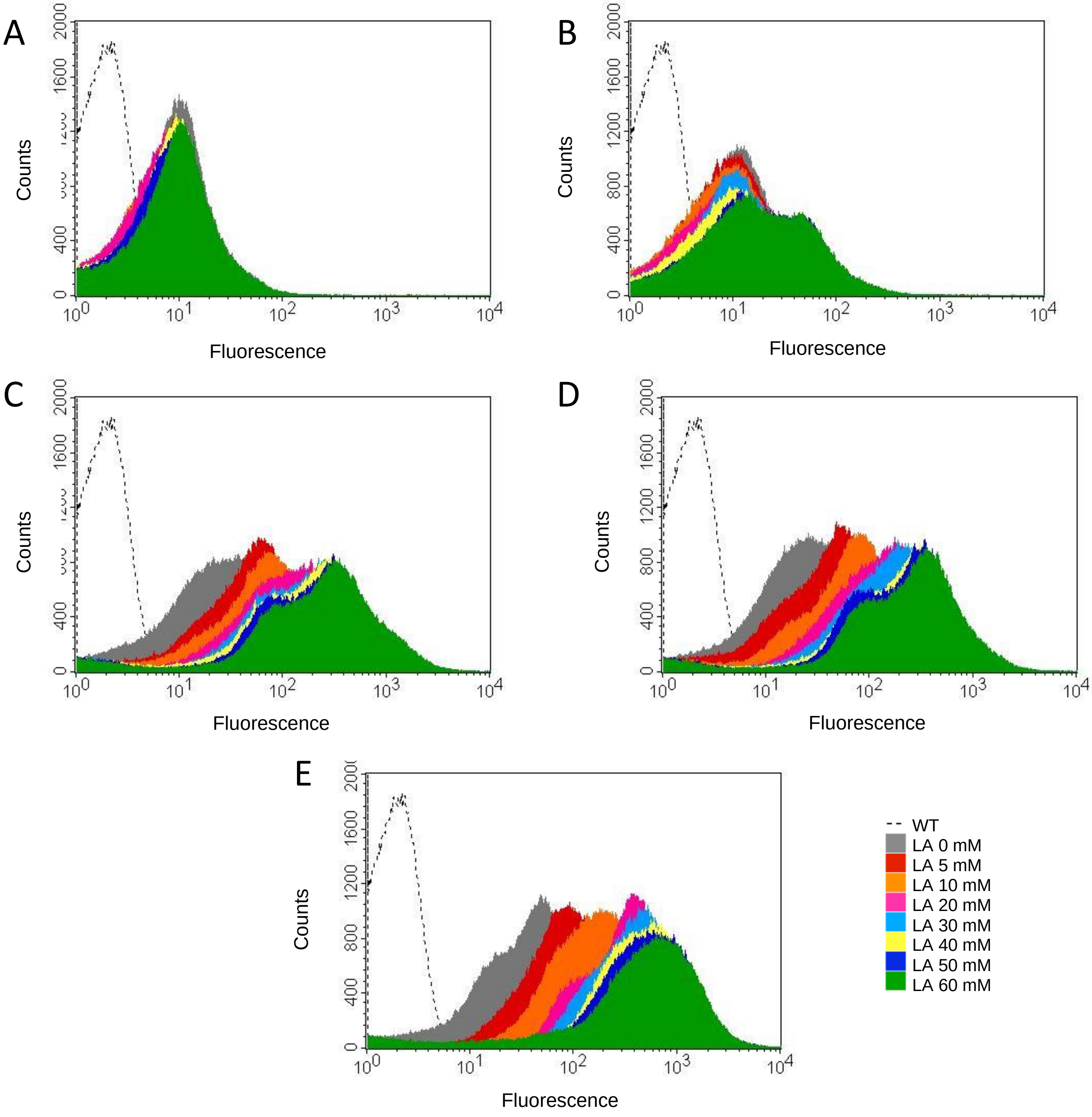

Supplementary Figure S3: **Flow cytometric analysis of UTR variants.** The recombinant strains IRF^{10f} (A), IRF^{100f} (B), IRF^{250f} (C), IRF^{500f} (D), IRF^{750f} (E), and IRF^{1Kf} (F) were evaluated with different concentrations of LA (5 to 60 mM). Filled peaks represent the fluorescence of the strains induced by the LA. Dotted peak refers to the WT.
